# Aphasia or Neglect after Thalamic Stroke: The Various Ways They may be Related to Cortical Hypoperfusion

**DOI:** 10.3389/fneur.2014.00231

**Published:** 2014-11-19

**Authors:** Rajani Sebastian, Mara G. Schein, Cameron Davis, Yessenia Gomez, Melissa Newhart, Kenichi Oishi, Argye E. Hillis

**Affiliations:** ^1^Department of Neurology, Johns Hopkins University School of Medicine, Baltimore, MD, USA; ^2^Department of Radiology, Johns Hopkins University School of Medicine, Baltimore, MD, USA; ^3^Department of Physical Medicine and Rehabilitation, Johns Hopkins University School of Medicine, Baltimore, MD, USA; ^4^Department of Cognitive Science, Johns Hopkins University, Baltimore, MD, USA

**Keywords:** acute thalamic stroke, aphasia, neglect, cortical hypoperfusion, diaschisis

## Abstract

Although aphasia and hemispatial neglect are classically labeled as cortical deficits, language deficits or hemispatial neglect following lesions to subcortical regions have been reported in many studies. However, whether or not aphasia and hemispatial neglect can be caused by subcortical lesions alone has been a matter of controversy. It has been previously shown that most cases of aphasia or hemispatial neglect due to acute non-thalamic subcortical infarcts can be accounted for by concurrent cortical hypoperfusion due to arterial stenosis or occlusion, reversible by restoring blood flow to the cortex. In this study, we evaluated whether aphasia or neglect occur after acute thalamic infarct without cortical hypoperfusion due to arterial stenosis or occlusion. Twenty patients with isolated acute thalamic infarcts (10 right and 10 left) underwent MRI scanning and detailed cognitive testing. Results revealed that 5/10 patients with left thalamic infarcts had aphasia and only 1 had cortical hypoperfusion, whereas 2/10 patients with right thalamic infarcts had hemispatial neglect and both had cortical hypoperfusion. These findings indicate that aphasia was observed in some cases of isolated left thalamic infarcts without cortical hypoerfusion due to arterial stenosis or occlusion (measured with time-to-peak delays), but neglect occurred after isolated right thalamic infarcts only when there was cortical hypoperfusion due to arterial stenosis or occlusion. Therefore, neglect after acute right thalamic infarct should trigger evaluation for cortical hypoperfusion that might improve with restoration of blood flow. Further investigation in a larger group of patients and with other imaging modalities is warranted to confirm these findings.

## Introduction

The role of subcortical structures in cognitive processing remains somewhat elusive. Over the last decades, the advent of functional imaging has led to a better understanding of the role of subcortical structures in cognitive processing. The thalamus has been of particular interest to researchers given that it projects to all areas of the neocortex including those areas in the frontal, temporal, and parietal regions that are commonly associated with language and cognition. Many functional imaging studies of language reveal thalamic participation in a variety of tasks and processes (1–10).

A recent study reviewed the role of the thalamus in 50 functional imaging studies of language tasks (11). The author found that thalamic activation was most commonly associated with generation tasks and naming; and the thalamic activation was seen bilaterally, left greater than right, along with activation in frontal and temporal cortical regions. Left parietal activation was seen in few studies. The peaks of activation loci were seen in all thalamic nuclei, with a bias toward left-sided and midline activation. The results of this literature review suggest that the thalamus may play a role in processes that involve the manipulation of lexical information, and that thalamic activation may be modulated by the difficulty of task demands. Similar results are reported in the clinical literature. Aphasia after left thalamic damage has also reported in numerous studies (4, 12–22). Further, language and cognitive deficits caused by stereotactic surgery (e.g., thalamotomy) or electric stimulation of the thalamus provided additional information about the role of specific thalamic nuclei in language and cognitive processes underlying language tasks (23–26).

In contrast to the large corpus of evidence that suggests involvement of the thalamus in language functions, the role of the thalamus in visuo-spatial processing, attention, and perception appears more obscure (27–34). A recent meta-analysis review explored the cognitive, affective, and behavioral disturbances following vascular thalamic lesions (35). The authors reviewed a study corpus of 465 patients with vascular thalamic lesions published in the literature since 1980 and found that 42 out of 465 (9%) cases had isolated thalamic lesions. Most of the cases reported pertained to language deficits after left thalamic lesion. Of patients with isolated thalamic lesions who were tested, 38.5% were impaired in spatial attention, 43.8% were impaired in comprehension, and 72.2% were impaired in naming. This review clearly highlights the importance of thalamus in higher-order behavioral functions; however, the mechanism that accounts for aphasia and neglect after thalamic lesion is still debated.

Several hypotheses have been advanced to explain the role of the thalamus in higher-order behavior (36). Nadeau and Crosson described five potential mechanisms associated with lesions of the thalamus: (1) direct impact of the thalamic lesion indicating that the thalamus is a crucial component of the cerebral network underlying neurocognitive processing; (2) diaschisis, or functional depression of regional neuronal metabolism and cerebral blood flow in anatomically connected cortical regions following dysfunction of the lesioned thalamus; (3) occlusion or stenosis of large cerebral vessels independently causing a thalamic stroke and hypoperfusion of the cortex; (4) cortical infarcts not detected by imaging; or (5) impaired “release” of language segments formulated by cortical regions into output. A sixth potential mechanism is indicated by animal studies showing alterations in cortical excitability in areas distant to the infarct that may be mediated by NMDA-dependent processes, spreading depression, or inflammation (37), rather than only anatomical disconnection. Diminished neuronal activity in an area remote to the infarct is the broadest meaning of “diaschisis.” Although the original concept proposed by Von Monakow (38) required an anatomical disconnection, the concept has developed recently to include a number of different forms of diaschisis, only some of which involve structural disconnection [see in Ref. (39) for review].

Baron and colleagues provided early evidence that deficits seen after thalamic lesion are secondary to cortical dysfunction due to diaschisis (40). They reported that, in patients with thalamic lesions, the magnitude of cognitive impairment was positively correlated with the degree of ipsilateral cortical hypometabolism demonstrated by PET [see also in Ref. (22)]. In previous studies, Baron and colleagues (41) also demonstrated that suppressed synaptic activity associated with reduction in metabolic demand in areas of diaschisis results in a reduction in cerebral blood flow. This spared neurovascular coupling in diaschisis was demonstrated by evaluating cerebral blood flow in regions of reduced glucose and oxygen metabolism. Since then, many others have demonstrated aphasia or neglect after thalamic lesions associated with cortical dysfunction attributable to diaschisis (35). In these cases, cortical dysfunction is demonstrated by measuring cortical metabolism or regional cerebral blood flow. Another study demonstrated a strong association between lesions in the right superior longitudinal fasciculus (SLF II) and left spatial neglect in a group study, and showed that right thalamic stroke was associated with chronic left neglect *only* when SLF II was damaged (42). This study provided evidence for the importance of thalamocortical connections (particularly to frontoparietal cortex) in the pathogenesis of chronic neglect.

However, some cases of aphasia or hemispatial neglect due to acute subcortical infarcts the associated cortical hypoperfusion cannot be attributed to diaschisis. Rather, the concurrent cortical hypoperfusion has been attributed to vascular stenosis or occlusion and seems to be responsible for the aphasia or neglect accounted in these cases (43, 44). In the one study, subcortical infarctions with aphasia and neglect were consistently associated with cortical hypoperfusion (in the middle cerebral artery territory), and reversal of the cortical hypoperfusion by restoring blood flow was associated with immediate resolution of their cortical deficits. Because reperfusion of the cortex, with the persistence of the subcortical infarct, would not have altered diaschisis or other potential mechanisms, the aphasia that resolved must have been due to hypofused, dysfunctional tissue that recovered function with reperfusion (44). Tissue that is receiving enough blood to survive but not enough to function, and recovers function by restoring blood flow, is the original meaning of “penumbra” (45). Penumbra has more recently come to mean tissue that will progress to infarct if blood flow is not restored [see in Ref. (46), this issue]. Therefore, at least some cases of aphasia due to subcortical infarcts are due to “penumbra” as originally defined – what we will refer to as “hypoperfusion due to arterial stenosis or occlusion”).

The cortical hypoperfusion associated with subcortical aphasia and neglect in the study by Hillis and colleagues (44) did not reflect diaschisis, for two reasons. First, cortical hypoperfusion was measured with dynamic contrast perfusion-weighted imaging (PWI) time-to-peak (TTP) maps, using a threshold of >2.5 s delay (although all had areas of cortical hypoperfusion with >4 s delay as well). While hypoperfusion due to diaschisis can occasionally be visualized (e.g., in cases of crossed cerebellar diaschisis due to large hemispheric strokes), the average delay in areas of diaschisis is 2 s or less, and does not correlate with measures of diaschisis obtained with PET (47, 48). Secondly, as noted, both the deficits and the hypoperfusion were reversed immediately with restored blood flow to the cortex (via stenting, urgent endarterectomy, or blood pressure elevation). Reperfusion did not alter the infarct; nor would it have altered diaschisis caused by the infarct.

It seems clear that thalamic lesions can cause aphasia or neglect by diaschisis. It is less clear how often aphasia or neglect after thalamic stroke is due cortical hypoperfusion caused by cortical stenosis or occlusion. The cases of reperfusion in the Hillis et al. study (44) described above included no thalamic lesions, so that study did not shed light on whether thalamic aphasia or neglect signals the presence of cortical hypoperfusion requiring restoration of blood flow. One reason that an answer to this question would be clinically useful is that it could be helpful in guiding acute intervention. For example, if the cognitive sequelae are frequently caused by cortical hypoperfusion due to stenosis or occlusion (the original meaning of penumbra), then acute intervention should focus on identifying marginally perfused tissue and restoring blood flow to improve function.

We hypothesized that some cases of thalamic infarction cause aphasia or neglect (directly or due to diaschisis) and other cases of thalamic infarction are associated with cortical hypoperfusion due to large vessel stenosis or occlusion that causes the aphasia or neglect. In the latter cases, cortical hypoperfusion can be due to a single plaque in the posterior cerebral artery (PCA) that both causes the infarct (by occluding a small branch to the thalamus) *and* causes cortical hypoperfusion of the larger PCA cortical territory. It is both scientifically and clinically important to determine if both of these mechanisms lead to thalamic aphasia or neglect. From a cognitive neuroscience standpoint, it is important to determine the role of the thalamus in language and spatial processing. From a clinical standpoint, it is important to determine whether aphasia or neglect in cases of thalamic stroke indicate the presence of hypoperfused tissue due to arterial stenosis or occlusion, such that function might be restored by reperfusion. Often perfusion imaging is not available, so that neurologists depend on the concept of a “diffusion-clinical mismatch” to guide urgent clinical decisions (49). That is, if a small stroke on diffusion-weighted imaging (DWI) cannot account for the clinical deficit, the patient is assumed to have marginally perfused tissue that accounts for the deficits, and would benefit from reperfusion. It is not clear whether or not a thalamic stroke on DWI, with aphasia or neglect represents a “diffusion-clinical mismatch.” In the current study, we determined the extent to which aphasia and hemispatial neglect with infarcts restricted to the thalamus could be accounted for by concurrent cortical hypoperfusion by studying patients with acute stroke with DWI and PWI TTP maps (to show cortical hypoperfusion due to arterial stenosis or occlusion) and detailed cognitive testing. We did not attempt to distinguish whether deficits in the absence of cortical hypoperfusion due to arterial stenosis or occlusion were due to the lesion itself or due to diaschisis.

## Materials and Methods

### Participants

A series of 20 participants with a first acute ischemic stroke, limited to the thalamus were recruited from the Johns Hopkins Hospital, Baltimore for this study. All participants were admitted and received MRI within 24 h of symptom onset. Additional exclusion criteria were as follows: (i) contraindication for MRI (e.g., implanted ferrous metal, claustrophobia); (ii) allergy to Gadolinium; (iii) hemorrhage on initial CT or MRI; (iv) impaired arousal or agitation requiring ongoing sedation; and (v) history of global intellectual deterioration (e.g., dementia); (vi) uncorrected visual acuity or hearing acuity. All participants gave informed consent (if they demonstrated intact comprehension), or their closest relative or legal representative consented (if they had impaired comprehension) to the study according to the Human Subjects Protocol. The study was approved by the Johns Hopkins University Institution Review Board.

A subset of 10 participants had isolated left thalamic infarct (infarct limited to the thalamus); (6 men and 4 women) and 10 participants had isolated right thalamic lesions (8 men and 2 women). The age of the participants ranged from 32 to 57 years, with a mean of 45.3 years (SD = 8.8) for those with isolated left thalamic lesions and from 35 to 68 years, with a mean of 52.3 years (SD = 9.8) for those with isolated right thalamic lesions. We were unable to determine which thalamic nuclei were affected due to the low spatial resolution of the DWI scans relative to thalamic nuclei. Therefore, the patients were grouped according to which thalamic artery was involved: tuberothalamic (polar); paramedian (thalamoperforator); inferior lateral (thalamogeniculate); and posterior choroidal (32, 50). MRI scans and testing was obtained after any acute treatment; two patients received IV tPA.

### Imaging protocol

MRI scans were obtained within 24 h from admission to the hospital. Participants had T2, fluid attenuation inversion recovery (FLAIR; to evaluate for old lesions), susceptibility weighted images (to evaluate for hemorrhage), PWI (to evaluate for areas of hypoperfusion), DWI (to evaluate for acute ischemia), MR angiography (to evaluate for stenosis, occlusion, aneurysm). DWI and PWI scans were 5 mm in thickness and provided whole-brain coverage. The total scan time lasted for approximately 30 min.

To measure the volume of infarct for each patient, a threshold of >30% intensity increase from the unaffected area in the DWI was applied, and a neurologist (KO), blinded to the behavioral data, manually modified the boundary of the thresholded area to remove false-positive and false-negative areas on RoiEditor^1^ (51). Ten randomly selected images were used to test intra and inter-operator reproducibility of this method. The intraclass correlation coefficient (ICC) was used to evaluate consistency of infarct volumes. Intra- and inter-observer reliability were excellent; the ICC was 0.98 for both within and across observers.

Areas of hypoperfusion in PWI were determined with TTP maps, using ImageJ^2^. TTP maps and DWI were co-registered with T2, which have better spatial resolution. The presence or absence of cortical hypoperfusion was identified by a trained technologist and neurologist blinded to the results of language testing. Regions of hypoperfusion were delineated by analysis of 20 color TTP maps. Hypoperfusion was defined as >4 s mean delay in TTP arrival of contrast across voxels in the region of interest (ROI) relative to the homologous region in the non-ischemic hemisphere. This threshold was selected because it corresponds to dysfunctional tissue defined in our previous studies and defined by PET (52, 53). For both left and right hemisphere stroke patients, we examined the entire cortex within the territory of the middle cerebral artery and PCA, as aphasia and neglect have been reported in association with lesions in each territory. The area of apparent hypoperfusion was segmented and served as the ROI. A mirror image of that ROI was drawn on the opposite hemisphere in the homologs region to compare the mean TTP. White matter hyperintensities were rated using the Cardiovascular Health Study (CHS) rating scale (ranging from 0 to 9, with 9 being “most extensive”). 0 was considered “none”; scores of 1–3 were considered “mild”; 3–6 “moderate,” and 7–9 “severe.”

### Test battery

All participants received testing within 48 h from admission to the hospital. Cognitive testing was completed after MRI scans. Participants with left thalamic stroke were administered a set of lexical tasks with stimuli matched for length (all stimuli), as well as for frequency and word class (for words). The tasks included (a) oral naming of black and white pictures [(54); *n* = 17]; (b) oral naming of objects with tactile input (*n* = 17); (c) oral reading of words (*n* = 34) and pseudowords (*n* = 25); and (d) auditory word comprehension using word/picture verification tasks (with 17 items, each presented once with the correct match, once with a semantically related foil, and once with a phonologically related foil). Please see in Ref. (44) for details.

Patients with right hemisphere stroke were given a battery of bedside tests to evaluate for hemispatial neglect at various levels of spatial representation. The tasks included the following:

(a) Perceptual tasks in which 30 circles and 30 circles with gaps are presented. Ten circles have a gap on the left side; 10 have a gap on the right; and 10 have no gap. The participant is asked to circle all the complete circles and draw an *X* over circles with a gap (55). Two forms of the task were presented: once with large circles, one with small circles.
(b) Perceptual motor tasks, including: line bisection, clock drawing, and copying the “Ogden scene” (a house, a fence and two trees).
(c) Oral reading and oral spelling of lists of frequency-matched and length-matched words and pseudowords. Only errors restricted to the contralesional (left) half of the word (e.g., sand read or spelled as “hand” or “and”) were scored as neglect errors.
(d) Motor extinction test, in which patients without hemiplegia were asked to click a golf counter with each hand, as quickly as possible for 1 min. The clicking rate was tested in three conditions: (i) each hand independently; (ii) the two hands simultaneously, with the hands at the subject’s sides; and (iii) the two hands simultaneously, with arms crossed across the chest (to distinguish impaired clicking with the left relative to the right hand versus impaired clicking on the left versus the right side of the midsagittal plane of the body (28).

Norms were obtained for the language and neglect battery by administering each battery to 46 volunteer control subjects who were awaiting surgical repair of unruptured intracerebral aneurysms or awaiting cardiac bypass surgery. Mean scores for each subtest ranged from 98.0% (SD = 3.1) correct in oral reading to 100% (SD = 0) correct in tactile naming. Abnormal performance was defined as 89% correct or lower; normal performance was defined as 90% correct or higher. This cut-off was selected because 89% was 3 SD below the mean on the subtest with the lowest mean. No control subject scored below 90% correct on any subtest of the battery. Participants were considered to have aphasia or neglect if they scored below 90% correct on any one or more of the subtests.

### Data analyses

Volume of infarct and hypoperfusion was measured as described above, without knowledge of language or neglect scores. The association between aphasia or hemispatial neglect and cortical hypoperfusion due to arterial stenosis or occlusion was evaluated by chi-square tests. Correlations between aphasia or neglect scores and volume of thalamic lesion and volume of hypoperfusion were evaluated by Spearman’s rank correlation.

## Results

Patient characteristics are given in Tables 1 and 2.

**Table 1:** **Demographic information for left thalamic infarct patients**.

ID	Age/sex	Education (years)	Lesion volume (cm^3^)	Hypoperfusion volume (cm^3^)	Naming % error	Auditory comprehension % error
**PATIENTS WITH APHASIA**						
1	57/M	12	4.256	2.6	12^a^	47^a^
2	37/M	12	4.091	–	35^a^	0
3	50/F	12	2.668	–	20^a^	41^a^
4	38/M	16	0.420	–	46^a^	5
5	39/F	12	0.879	–	46^a^	0
**PATIENTS WITHOUT APHASIA**						
6	32/M	12	1.243	–	0	6
7	51/F	12	0.738	–	3	10
8	41/M	12	0.382	3.71	6	0
9	55/F	12	0.409	–	0	0
10	53/M	16	0.822	–	0	0

*^a^Evidence of aphasia*.

**Table 2:** **Demographic information for right thalamic infarct patients**.

ID	Age/sex	Education (years)	Lesion volume (cm^3^)	Hypoperfusion volume (cm^3^)	Copy scene % error	Line bisection % error
**PATIENTS WITH NEGLECT**						
11	68/M	5	0.418	2.07	78^a^	12^a^
12	61/M	12	0.827	6.27	15^a^	4
**PATIENTS WITHOUT NEGLECT**						
13	35/M	12	0.699	–	0	1.87
14	53/M	12	0.347	–	0	0.4
15	46/F	11	0.215	–	0	2.6
16	43/F	12	0.791	–	0	3.4
17	50/M	10	1.036	–	0	3.82
18	59/M	11	0.600	–	3	2.5
19	48/M	12	1.169	–	3	1.9
20	60/M	7	0.703	–	3	3.6

*^a^Evidence of neglect*.

### Aphasia and left thalamic lesion

**Figure 1:**
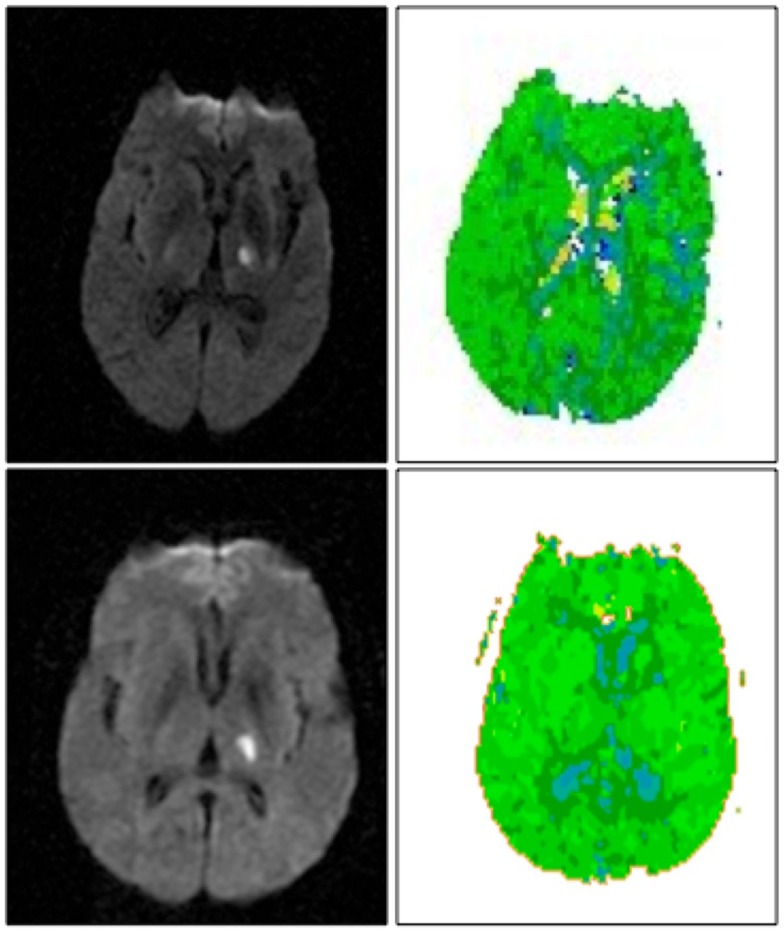
**Left thalamic infarct with cortical hypoperfusion associated with cortical hypoperfusion (top) and without cortical hypoperfusion (bottom)**. DWI scans are shown on the left, PWI scans are shown on the right. Scans are in radiological convention (left hemisphere on right). Blue areas are hypoperfused.

Of the10 patients with isolated left thalamic lesions, 5 had aphasia (see Table 1 for language scores). Four of the five aphasic patients had normal cortical perfusion. All patients with aphasia had fluent speech. Three had isolated naming deficits with normal comprehension/repetition; all of these had normal cortical perfusion (Figure 1, top). Another two patients had both naming impairment and auditory comprehension impairment, and one of these patients had cortical hypoperfusion due to arterial stenosis (Figure 1, bottom). Cortical hypoperfusion was seen in the MCA and/or PCA territory (e.g., inferior temporal cortex) in areas reported to be important for language, in all cases with aphasia.

Five patients with left thalamic lesions did not have aphasia; and four of these patients had normal cortical perfusion. One patient with no language deficit had cortical hypoperfusion. The hypoperfusion was in the left parietal cortex. Other associated neurological deficits in patients with left thalamic lesions included right-sided weakness, numbness, and dysarthria.

There was no association between the presence of left cortical hypoperfusion and either naming impairment (*X*^2^ = 0.0, *p* = 1) or auditory comprehension impairment (*X*^2^ = 1.4, *p* = 0.24). Further, there was no significant correlation between volume of thalamic infarct and severity of the naming impairment (ρ = −0.098; *p* = 0.79) or severity of comprehension impairment (ρ = −0.084; *p* = 0.82).

### Hemispatial neglect and right thalamic lesion

**Figure 2:**
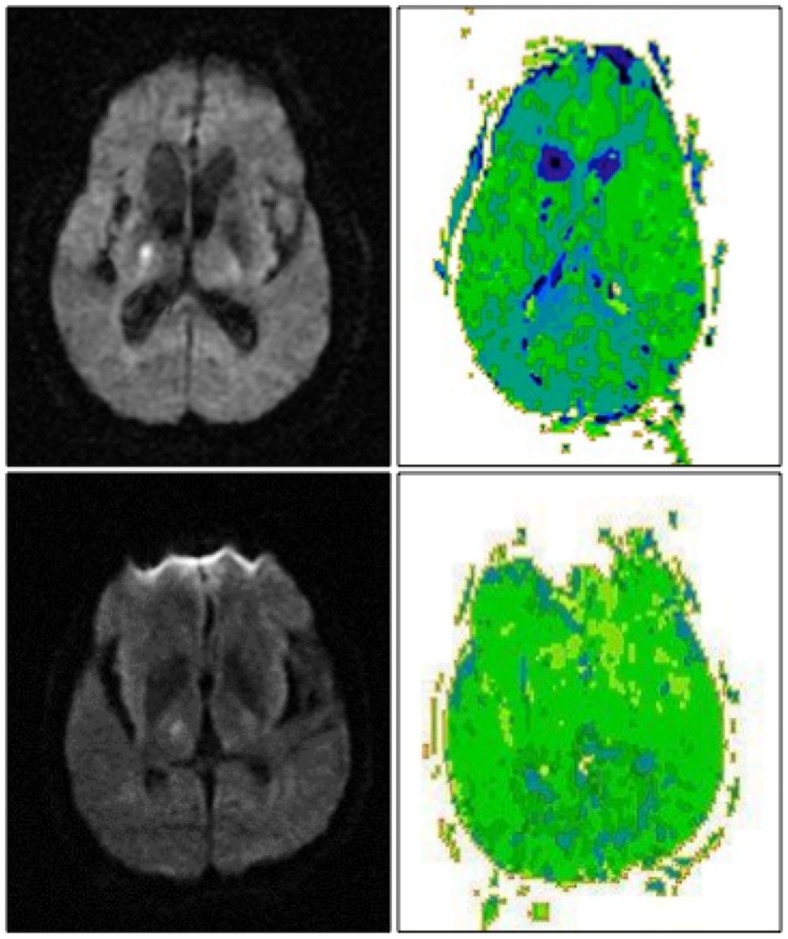
**Right thalamic infarct with cortical hypoperfusion and associated neglect (top) and without cortical hypoperfusion and no associated neglect (bottom)**. DWI scans are shown on the left, PWI scans are shown on the right. Scans are in radiological convention (right hemisphere on left). Blue/darker green areas are hypoperfused.

Of the10 patients with exclusively right thalamic lesions, two had left hemispatial neglect. Both the patients had cortical hypoperfusion (Figure 2, top). The area of hypoperfusion included inferior temporal/fusiform cortex, which has been associated with hemispatial neglect (56). Eight patients without hemispatial neglect showed no cortical hypoperfusion (Figure 2, bottom) (*X*^2^ = 10; *p* = 0.001). Other associated neurological deficits in patients with right thalamic lesions included left-sided weakness, numbness, and dysarthria.

Severity of neglect measured by deviation to the right on line bisection task correlated with volume of cortical hypoperfusion (ρ = 0.67; *p* = 0.02), but did not correlate with volume of thalamic lesion (ρ = −0.26; *p* = 0.65).

### Lesion location

As is the case for most thalamic strokes (50), the majority of lesions were in the distribution of the inferior lateral (thalamogeniculate) artery, which arises from the PCA (Tables 3 and 4). There were too few patients with white matter intensities (three with left thalamic lesions, one with right thalamic lesion, mostly mild) to determine any association with neglect or aphasia (Tables 3 and 4).

**Table 3:** **Lesion characteristics for patients with left thalamic stroke**.

ID	Large vessel stenosis/occlusion	Arterial territory of infarct	White matter changes	Lesion volume (mm^3^)	Hypoperfusion volume (cm^3^)	Naming % error	Auditory comprehension % error
**PATIENTS WITH APHASIA**							
1	Left PCA occlusion	Inferior lateral (thalamogeniculate)	Mild	4.256	2.6	12^a^	47^a^
2	No stenosis	Inferior lateral (thalamogeniculate)	None	4.091	–	35^a^	0
3	No stenosis	Tuberothalamic (polar)	None	2.668	–	20^a^	41^a^
4	No stenosis	Posterior choroidal	Mild	0.420	–	46^a^	5
5	No stenosis	Inferior lateral (thalamogeniculate)	None	0.879	–	46^a^	0
**PATIENTS WITHOUT APHASIA**							
6	Left MCA and PCA stenosis	Inferior lateral (thalamogeniculate)	None	1.243	–	0	6
7	No stenosis	Inferior lateral (thalamogeniculate)	Mild	0.738	–	3	10
8	Midbasilar and left PCA stenosis	Inferior lateral (thalamogeniculate)	None	0.382	3.71	6	0
9	No stenosis	Inferior lateral (thalamogeniculate)	None	0.409	–	0	0
10	No stenosis	Posterior choroidal	None	0.822	–	0	0

*^a^Evidence of aphasia*.

**Table 4:** **Lesion characteristics for patients with right thalamic stroke**.

ID	Large vessel stenosis/occlusion	Arterial territory of infarct	White matter changes	Lesion volume (cm^3^)	Hypoperfusion volume (cm^3^)	Copy scene % error	Line bisection % error
**PATIENTS WITH NEGLECT**							
11	Severe right PCA stenosis	Inferior lateral (thalamogeniculate)	None	0.418	2.07	78^a^	12*
12	Severe right PCA stenosis; right M1 stenosis	Inferior lateral (thalamogeniculate)	None	0.827	6.27	15^a^	4
**PATIENTS WITHOUT NEGLECT**							
13	Left vertebral artery dissection	Posterior choroidal	None	0.699	–	0	1.87
14	No stenosis	Inferior lateral (thalamogeniculate)	None	0.347	–	0	0.4
15	No stenosis	Posterior choroidal	None	0.215	–	0	2.6
16	No stenosis	Posterior choroidal	Mild	0.791	–	0	3.4
17	Congenitally absent right vertebral artery	Inferior lateral (thalamogeniculate)	None	1.036	–	0	3.82
18	Mild (30–40%) left ICA stenosis	Inferior lateral (thalamogeniculate)	None	0.600	–	3	2.5
19	Mild right PCA and left MCA stenosis	Posterior choroidal	None	1.169	–	3	1.9
20	No stenosis	Posterior choroidal	None	0.703	–	3	3.6

*^a^Evidence of neglect*.

## Discussion

The goal of the present study was to evaluate whether acute thalamic aphasia or neglect can be caused by cortical hypoperfusion due to large vessel stenosis or occlusion. We also wished to determine the frequency of this mechanism relative to that of a direct thalamic cause or diaschisis (presumably via disruption of subcortical–cortical circuits). We were not able to evaluate diaschisis in this study, using TTP to study perfusion (47). Our study indicates that cortical hypoperfusion due to large vessel stenosis or occlusion cannot adequately explain language deficits in patients with acute left thalamic stroke. On the other hand, cortical hypoperfusion due to arterial stenosis may account for, or at least contribute substantially to, hemispatial neglect in acute right thalamic stroke.

Our results from patients with left thalamic lesions are consistent with the findings of several studies showing that the language deficits that frequently follow isolated thalamic strokes may be caused by dysfunction of the thalamic-cortical system via diaschisis (36, 40, 57). This proposal assumes that the loss of input from the thalamus directly causes the cortical dysfunction, which secondarily results in mild diffuse hypoperfusion detectable by SPECT or PET because of spared neurovascular coupling (19). For example, in the Radanovic study of five patients with left thalamic stroke, there was a correspondence between cortical hypoperfusion and the naming impairments (present in three of five patients) and comprehension impairments (present in four of five patients), suggesting that the thalamus’s participation in language is made through its influence on the cortex.

The proposed account of diaschisis does not fully explain the results in patients with right thalamic lesions. Only 2 out of the 10 patients had hemispatial neglect, and both the patients had cortical hypoperfusion as seen on PWI TTP maps (>4 s delay). This degree of delay is not a reflection of decreased metabolic demand, but caused by large vessel stenosis or occlusion (confirmed by MRA in these cases). Furthermore, the severity of neglect correlated with volume of hypoperfusion, not volume of infarct. Because we had only 10 patients, we cannot conclude that hemispatial neglect occurs *only* when there is cortical hypoperfusion caused by arterial stenosis. In fact, other studies indicate that at least chronic neglect occurs only when there is disruption of thalamocortical white matter tracts (42). However, cortical hypoperfusion due to arterial stenosis or occlusion (that is independent of the thalamic infarct) does seem to have a significant role in the development of marked hemispatial neglect in acute stroke. The thalamic lesions in patients with neglect mainly involved the inferior lateral (thalamogeniculate) territory, which supplies the ventroposerior medial (VPM) and ventroposterior lateral (VPL) nuclei, and only a small portion of the ventrolateral nucleus (32). As VPM and VPL are not known to have a role in attention, neglect can in these case can be explained by cortical hypoperfusion due to plaque in the right PCA (which also occluded the inferior lateral artery as it branched off the PCA, which caused the thalamic infarct).

The relatively young age of our stroke participants may also have influenced the results. The mean age of the participants with right thalamic stroke in this study was 52.3 years. Gottesman et al. (58) found that among patients with acute right hemispheric stroke, neglect occurs at higher frequency and at increasing severity in older patients (above 65 years). This age effect is independent of the size of the stroke and the severity of other presenting clinical symptoms. Similarly, Ringman et al. (59) found that older patients experience neglect at higher rates than younger patients. Thus, our participants had a lower risk of hemispatial neglect due to relatively young age. Perhaps, only those relatively young stroke patients with large vessel stenosis and severe cortical hypoperfusion had neglect; while older patients with thalamic lesions without severe cortical hypoperfusion (but only cortical diaschisis) would have neglect. Likewise, increased burden of white matter disease is associated with increased severity of neglect (60); but only one patient with right thalamic stroke had mild white matter disease, perhaps because of the young age of our population.

Is it plausible that both hypoperfusion due to arterial stenosis and diaschisis co-exist in some cases. Reperfusion of the cortex is one way to disentangle these mechanisms. Restoring blood flow to the cortex eliminates hypoperfusion caused by arterial stenosis, but would not affect diaschisis (because the thalamic lesion is still present). Future studies of thalamic stroke before and after intervention to restore blood flow to hypoperfused cortex would be useful.

In summary, the present study indicates that some cases of neglect after thalamic stroke are at least partially due to cortical hypoperfusion caused by arterial (PCA) stenosis or occlusion. Therefore, hemispatial neglect in the presence of an infarct restricted to the thalamus should raise the suspicion for presence of marginally perfused tissue that might recover function if reperfused, and perhaps the need for perfusion or vessel imaging and intervention. In contrast, we were not able to identify such a mechanism in thalamic aphasia. Rather, our results provide additional evidence that the left thalamus has a direct role in language processing (at least) by activating cortical areas involving specific tasks. The small size of our patient group demands further studies to support these findings. Multimodality imaging, including vessel imaging, diffusion tensor imaging, functional imaging studies, and measurement of rCBF would further clarify the roles of various potential mechanisms [including distinct types of diaschisis; (39)] of underlying thalamic aphasia and neglect.

## Conflict of Interest Statement

The authors declare that the research was conducted in the absence of any commercial or financial relationships that could be construed as a potential conflict of interest.

## Footnotes

^1^http://www.MRIstudio.org

^2^http://rsb.info.nih.gov.proxy1.library.jhu.edu/ij
